# Supplementary material for: Life-history trade-offs explain local adaptation in Arabidopsis thaliana
Source: eLife. 2026 Nov 10;14:RP107477. doi: 10.7554/eLife.107477 (PMC13750797; doi:10.7554/eLife.107477)
Supplement: Supplementary file 1 [file elife-107477-fig7-data1.pdf]

Figure 7—source data 1. Full anova table.

Model term	Df	Sum.Sq	Mean.Sq	F.value	*p*.value
exp	3	272.6529848	90.88432826	605.8932294	~0
year	1	93.69213929	93.69213929	624.6118988	1.27E-135
block	2	20.92749542	10.46374771	69.75805416	6.57E-31
id	199	247.4817467	1.243626868	8.290814418	4.83E-215
exp:year	3	422.6119606	140.8706535	939.1341372	~0
exp:block	6	38.96877675	6.494796124	43.29847702	7.91E-53
year:block	2	10.70577142	5.352885709	35.68576971	3.40E-16
exp:id	596	181.3343515	0.304252268	2.028340777	7.76E-42
year:id	199	97.24519958	0.488669345	3.257783306	7.53E-48
exp:year:block	6	21.92561215	3.654268692	24.36169911	6.54E-29
exp:year:id	582	129.670064	0.222800797	1.485333029	6.28E-13
Residuals	18604	2790.610558	0.150000568	NA	NA
