# Supplementary material for: Life-history trade-offs explain local adaptation in Arabidopsis thaliana
Source: eLife. 2026 Nov 10;14:RP107477. doi: 10.7554/eLife.107477 (PMC13750797; doi:10.7554/eLife.107477)
Supplement: Peer review history

# Editor report: eLife Assessment

**DOI:** 10.7554/eLife.107477.3.sa0

**Authors:** Bernhard Schmid

**Affiliations:** University of Zurich Switzerland

**Keywords:** Convincing, Important

This study provides **convincing** evidence that the genetics of local adaptation in Arabidopsis is shaped by fluctuations in the environment and interactions with genotype and location. This is an **important** dataset contributing to the developing understanding of non-linear selection in plants and beyond.

# Referee report: Reviewer #1 (Public review)

**DOI:** 10.7554/eLife.107477.3.sa1

[Editors' note: this version has been assessed by the Reviewing Editor without further input from the original reviewers. The authors have addressed the comments raised in the previous round of review both by revising their original presentation where necessary and by providing responses to the reviewers in cases where they disagreed that revision of the original presentation would be necessary.]

Summary:

As a general phenomenon, adaptation of populations to their respective local conditions is well-documented, though not universally. In particular, local adaptation has been amply demonstrated in *Arabidopsis thaliana*, the focal species of this research, which is naturally highly selfing. Here, the authors report assays designed to evaluate the spatial scale of fitness variation among source populations and sites, as well as temporal variability in fitness expression. Further, they endeavor to identify traits and genomic regions that contribute to the demonstrated variation in fitness.

Strengths:

With many (200) inbred accessions drawn from throughout Sweden, the study offers an unusually fine sampling of genetic variation within this much-studied species, and through assays in multiple sites and years, it amply demonstrates the context-dependence of fitness expression. It supports the general phenomenon of local adaptation, with multiple nuances. Other examples exist, but it is of value to have further cases illustrating not only the context-dependence of fitness expression but also the sometimes idiosyncratic nature of fitness variation. I commend the authors on their cautionary language in relation to inferences about the roles of particular genomic regions (e.g.l.140-144; l.227)

# Referee report: Reviewer #2 (Public review)

**DOI:** 10.7554/eLife.107477.3.sa2

Summary:

The goal of this study was to find evidence for local adaptation in survival and fecundity of the model plant *Arabidopsis thaliana*. The authors grew a large set of Swedish Arabidopsis accessions at four common garden sites in northern and southern Sweden. Accessions were grown from seed in trays, which were laid on the ground at each site in late summer, screened for survival in fall and the following spring, and fecundity was determined from rosette size and seed production in spring. Experiments were complemented by 'selection experiments', in which seeds of the same accessions were sown in plots, and after two years of growth, plants were sampled to determine fitness from genotype frequencies, providing a more comprehensive evaluation of lifetime fitness than can be gleaned from fecundity alone.

As the main result, southern accessions had higher mortality in northern sites in one of two years, but also suffered more slug damage in southern sites in one year, indicating a potential link between frost tolerance and herbivore resistance. Fecundity of accession was highest when growing close to the 'home' environment, but while accessions from one sand dune population in southern Sweden had among the lowest fecundities overall, they consistently had the highest fitness in the selection experiment. Accessions from this population had large seed size and rapid root growth, which might be related to establishment success when arriving in a new, partially occupied habitat. However, neither trait could fully explain the very high fitness of this population, suggesting the presence of other, unmeasured traits.

Overall, the authors could provide clear evidence of local adaptation in different traits for some of their experiments, but they also highlight high temporal and spatial variability that makes prediction of microevolutionary change so challenging.

Strengths:

A major strength of this study is the highly comprehensive evaluation of different fitness-related traits of Arabidopsis under natural conditions. The evaluation of survival and fecundity in common garden experiments across four sites and two years provides an estimate of variability and consistency of results. The addition of the 'selection experiment' provides an extended view on plant fitness that is both original and interesting, in particular highlighting potential limitations of 'fitness-proxies' such as seed production that don't take into account seedling establishment and competitive exclusion.

Throughout the study, the authors have gone to impressive depths in exploring their data, and particularly the discovery of 'native volunteers' in selection experiment plots and their statistical treatment is very elegant and has resulted in compelling conclusions. Also, while the authors are careful in the interpretation of their GWAS results, they nonetheless highlight a few interesting gene candidates that may be underlying the observed plant adaptations, and which likely will stimulate further research.

Overall, this study will likely make an important contribution to the field of evolutionary biology, and it is another very strong example of how the extensive molecular tools in Arabidopsis can be leveraged to address fundamental questions in evolution and ecology, to an extent that is not (yet) possible in other plant systems.

# Referee report: Reviewer #3 (Public review)

**DOI:** 10.7554/eLife.107477.3.sa3

Summary:

The manuscript presents a large common garden experiment across Sweden using solely local germplasm. Additionally, there is a collection of selection experiments that begin investigating the factors shaping fecundity in these populations. This provides an impressive amount of data and analysis investigating the underlying factors involved. Together, this helps support the data showing that fluctuations and interactions are key components determining Arabidopsis fitness and are more broadly applicable across plant and non-plant species.

Strengths:

The field trials are well conducted with extensive effort and sampling. Similarly while the genetic analysis is complex it is well conducted and reflects the complexity of dealing with population structure that may be intricately linked to adaptive structure. This has no real solution and the option of presenting results with and without correction is likely the only appropriate option.

# Author comment: Author response

**DOI:** 10.7554/eLife.107477.3.sa4

**Authors:** Benjamin Brachi, Daniele Filiault, Rahul Pisupati, Tal Dahan, Anna Igolkina, Alison Anastasio, Mathew S Box, Susan Duncan, Talia Karasov, Envel Kerdaffrec, Laura Merwin, Timothy C Morton, Viktoria Nizhynska, Polina Novikova, Fernando Rabanal, Takashi Tsuchimatsu, Torbjörn Säll, Caroline Dean, Svante Holm, Joy Bergelson, Magnus Nordborg

**Affiliations:** University of Chicago Chicago United States; Gregor Mendel Institute Vienna Austria; Gregor Mendel Institute Vienna Austria; Gregor Mendel Institute Vienna Austria; Gregor Mendel Institute Vienna Austria; University of Chicago Chicago United States; John Innes Centre Norwich United Kingdom; John Innes Centre Norwich United Kingdom; University of Utah Salt Lake City United States; University of Fribourg Fribourg Switzerland; University of Chicago Chicago United States; University of Chicago Chicago United States; Austrian Academy of Sciences Vienna Austria; Gregor Mendel Institute Vienna Austria; Gregor Mendel Institute Vienna Austria; Gregor Mendel Institute Vienna Austria; Lund University Lund Sweden; John Innes Centre Norwich United Kingdom; Mid-Sweden University Sundsvall Sweden; University of Chicago Chicago United States; Gregor Mendel Institute Vienna Austria

The following is the authors’ response to the original reviews.

**Public Reviews:**

**Reviewer #1 (Public review):**

Summary:

As a general phenomenon, adaptation of populations to their respective local conditions is well-documented, though not universally. In particular, local adaptation has been amply demonstrated in *Arabidopsis thaliana*, the focal species of this research, which is naturally highly selfing. Here, the authors report assays designed to evaluate the spatial scale of fitness variation among source populations and sites, as well as temporal variability in fitness expression. Further, they endeavor to identify traits and genomic regions that contribute to the demonstrated variation in fitness.

Strengths:

With many (200) inbred accessions drawn from throughout Sweden, the study offers an unusually fine sampling of genetic variation within this much-studied species, and through assays in multiple sites and years, it amply demonstrates the context-dependence of fitness expression. It supports the general phenomenon of local adaptation, with multiple nuances. Other examples exist, but it is of value to have further cases illustrating not only the context-dependence of fitness expression but also the sometimes idiosyncratic nature of fitness variation. I commend the authors on their cautionary language in relation to inferences about the roles of particular genomic regions (e.g.l.140-144; l.227)

Weaknesses:

To my mind, the manuscript is written primarily for the Arabidopsis community. This community is certainly large, but there are many evolutionary biologists who could appreciate this work but are not invited to do so. The authors could address the broader evolution community by acknowledging more of the relevant work of others (I've noted a few references in my comments to the authors). At least as important, the authors could make clearer the fact that *A. thaliana* is (almost) strictly selfing and how this feature of its biology both enables such a study and also limits inferences from it. Further, it seems to me that though I could be wrong, readers would appreciate a more direct, less discursive style of writing, and one that makes the broader import of the focal questions clearer.

We agree that connecting the paper better to the broader field is desirable, and have tried to do this in the revision, including adding a brief description of *A. thaliana* that mentions the fact that it is highly selfing. That the availability of inbred lines influences the study design is fairly obvious. As for the inferences, we would argue that our main point—“know your organism”—is universal. For example, our selection experiments and common garden experiments produced diametrically opposite results in terms of fitness, and we attribute this to the importance of seedling establishment. It is hardly surprising that seedling establishment matters for an annual plant that favors disturbed habitats, but this is probably true for both outcrossers and selfers (although it is probable that selfing itself is an adaptation to such habitats), and could well be true for some long-lived trees as well. The Devil is in the details.

As a reader, I would value seeing estimates of the overall fitness of the accessions in the different conditions, i.e., by combining the survival and fecundity results of the common garden experiments.

Combining estimates would be possible in the common garden experiments, and would bring us somewhat closer to total fitness estimates, although as noted by another reviewer (and also emphasized by us), the time scale of our experiment is not sufficient to evaluate the trade-off between survival and fecundity. Furthermore, we would still be missing the establishment component of fitness, which we found to be extremely important. Therefore little would be gained by combining the estimates, while at the same time losing resolution to disentangle the fitness components. We thus decided to focus on the individual fitness components and leave a qualitative consideration of their joint effect for the Discussion.

**Reviewer #2 (Public review):**

Summary:

The goal of this study was to find evidence for local adaptation in survival and fecundity of the model plant *Arabidopsis thaliana*. The authors grew a large set of Swedish Arabidopsis accessions at four common garden sites in northern and southern Sweden. Accessions were grown from seed in trays, which were laid on the ground at each site in late summer, screened for survival in fall and the following spring, and fecundity was determined from rosette size and seed production in spring. Experiments were complemented by 'selection experiments', in which seeds of the same accessions were sown in plots, and after two years of growth, plants were sampled to determine fitness from genotype frequencies, providing a more comprehensive evaluation of lifetime fitness than can be gleaned from fecundity alone.

To clarify, fecundity was determined from total plant area using photos of the mature stems, not the rosettes or direct counting of seeds. That said, it is true that our fecundity estimate was well correlated with rosette area. Furthermore, we validate our fecundity estimates by showing they were highly correlated with seed production estimated by measuring and counting siliques on a separate set of plants grown under common garden conditions in one of our sites (Brachi et al. 2022).

As the main result, southern accessions had higher mortality in northern sites in one of two years, but also suffered more slug damage in southern sites in one year, indicating a potential link between frost tolerance and herbivore resistance. Fecundity of accession was highest when growing close to the 'home' environment, but while accessions from one sand dune population in southern Sweden had among the lowest fecundities overall, they consistently had the highest fitness in the selection experiment. Accessions from this population had large seed size and rapid root growth, which might be related to establishment success when arriving in a new, partially occupied habitat. However, neither trait could fully explain the very high fitness of this population, suggesting the presence of other, unmeasured traits.

Another clarification: the small set of “beach” accessions that performed well in the selection experiments came from several beaches on the Baltic coast of Skåne, and we also had one such accession from the island of Gotland, 400 km away as the bird flies. Furthermore, we sampled measured seed size in several hundred additional accessions and demonstrated that large seeds are indeed characteristic of this habitat.

Overall, the authors could provide clear evidence of local adaptation in different traits for some of their experiments, but they also highlight high temporal and spatial variability that makes prediction of microevolutionary change so challenging.

Strengths:

A major strength of this study is the highly comprehensive evaluation of different fitness-related traits of Arabidopsis under natural conditions. The evaluation of survival and fecundity in common garden experiments across four sites and two years provides an estimate of variability and consistency of results. The addition of the 'selection experiment' provides an extended view on plant fitness that is both original and interesting, in particular highlighting potential limitations of 'fitness-proxies' such as seed production that don't take into account seedling establishment and competitive exclusion.

Throughout the study, the authors have gone to impressive depths in exploring their data, and particularly the discovery of 'native volunteers' in selection experiment plots and their statistical treatment is very elegant and has resulted in compelling conclusions. Also, while the authors are careful in the interpretation of their GWAS results, they nonetheless highlight a few interesting gene candidates that may be underlying the observed plant adaptations, and which likely will stimulate further research.

Overall, the authors provide a rich new resource that is relevant and interesting both in the context of general evolutionary theory as well as more specifically for molecular biology.

Weaknesses:

While the repetition of the common garden experiments over two years is certainly better than no repetition (hence its mention also under 'strengths'), the very high variability found between the two years highlights the need for more extensive temporal replication. In this context, two temporal replicates are the bare minimum, and more repeats in time would be necessary to draw any kind of conclusion about the role of 'high mortality' and 'low mortality' years for the microevolution of Arabidopsis. It also seems that the authors missed an opportunity to explore potentially causal variation among years, as they did not attempt to relate winter mortality to actual climatic variables, even though they discuss winter harshness as a potential predictor.

We agree that two years is insufficient to understand how variation in selective pressures compound over time to generate micro-evolutionary change. The eight-year data in Oakley et al. (2023), which we discuss in the paper, support this. Our results are nonetheless sufficient to demonstrate the idiosyncratic nature of selection. In the revision, we further emphasize that far longer time series would be needed for definitive conclusions on long-term micro-evolutionary change.

Our short time series is one reason why we do not try to correlate with climate data, as this would amount to doing statistics with four data points (mostly two groups of accession N vs S, with mostly homogenous climates within groups, and two years). Another reason is that we simply have no idea how the kind of meteorological data that are publicly available would affect plant performance on a local scale. Hence we make no claims..

The low temporal variation also makes the accidental slug herbivory appear somewhat random. Potted plants are notoriously susceptible to slug herbivory, and while it is certainly nice that slug damage predominantly affected one group of accessions, it nonetheless raises the question whether this reflects a 'real' selection pressure that plants commonly face in their respective local environments.

Characterizing the plants as “potted” is not accurate. A more serious objection is having what is effectively an *A. thaliana* monoculture. But indeed we have no idea whether slugs exert a significant selective pressure on *A. thaliana* in Sweden, and we make no claims to that effect. The evidence for selection on glucosinolates by generalist herbivores such as slugs is fairly strong, but the precise agent is not known, and probably varies over time and space. Our results merely demonstrate one possibility.

The addition of the 'selection experiment' is certainly original and provides valuable additional insights, but again, it seems a bit questionable which natural process really has affected this outcome. While the genetic and statistical analysis of this experiment seems to be state-of-the-art, the experimental design is rather rudimentary compared to more standard selection experiments. Specifically, the authors added seeds from greenhouse-grown mothers to experimental plots and only sampled plants two years later. This means that, potentially, the first very big bottleneck was germination under natural conditions, which may have already excluded many of the accessions before they had a chance to grow. While this certainly is one type of selection, it is not exactly the type of selection that a 2-year selection experiment is set up to measure. Either initially establishing the selection experiment from plants instead of seeds, or genotyping the population over several generations, would have substantially strengthened the conclusions that could be drawn from this experiment.

We 100% agree that more data would have been beneficial, and hence we do not make any claims about the nature of selection. The selection experiment was an experiment per se, and we were lucky that very large fitness differences turned out to exist. As for initial selection on dormancy “set” incorrectly in greenhouse-grown seeds, we agree that this is a possibility, but we do not think it is likely to explain the data for several reasons. First, why would these differences favor a small set of beach accessions over every other accession in four very different field sites? Second, existing dormancy estimates do not predict fitness in our selection experiments. Third, the same seed batches germinated uniformly in the common-garden experiments with minimal stratification. Fourth, a much simpler explanation—seed size—exists. We clarify this in the revision while retaining our original message that further experiments are needed (and are underway).

Also, the complete lack of information on population density is a bit problematic. It is not clear if there were other (non-Arabidopsis) plants present in the plots, how many Arabidopsis plants were established, if numbers changed over the year, etc. Given all of these limitations, calling this a 'selection experiment' is in fact somewhat misleading.

Seeds were introduced into sites that appeared appropriate for *A. thaliana*, leaving the background community intact. We provided information on sowing density; the density of plants (*A. thaliana* and other species) that we obtained during the course of the experiments varied considerably between sites, much like in natural populations, although we lack systematic measurements. We provide more information (including photos) in the revision.

Despite these weaknesses, the authors could achieve their main goals, and despite the somewhat minimal temporal replication, they were lucky to sample two fairly distinct years that provided them with interesting variation, which they could partially explain using the variation among their accessions. Overall, this study will likely make an important contribution to the field of evolutionary biology, and it is another very strong example of how the extensive molecular tools in Arabidopsis can be leveraged to address fundamental questions in evolution and ecology, to an extent that is not (yet) possible in other plant systems.

**Reviewer #3 (Public review):**

Summary:

The manuscript presents a large common garden experiment across Sweden using solely local germplasm. Additionally, there is a collection of selection experiments that begin investigating the factors shaping fecundity in these populations. This provides an impressive amount of data and analysis investigating the underlying factors involved. Together, this helps support the data showing that fluctuations and interactions are key components determining Arabidopsis fitness and are more broadly applicable across plant and non-plant species.

Strengths:

The field trials are well conducted with extensive effort and sampling. Similarly while the genetic analysis is complex it is well conducted and reflects the complexity of dealing with population structure that may be intricately linked to adaptive structure. This has no real solution and the option of presenting results with and without correction is likely the only appropriate option.

Weaknesses:

A significant finding from this study was that fecundity is shaped more by yearly fluctuations and their interaction with genotype than it is by the main effect of location or genotype. Another significant finding is that the strength of selection can be quite strong, with nearly 5x ranges across accessions. It should be noted that there are a number of other studies using Arabidopsis in the wild with multiple years and locations that found similar observations beyond the Oakley citation. In general, the context of how these findings relate to existing knowledge in Arabidopsis is a bit underdeveloped.

We have tried to remedy this in the revision (see also comments by Reviewer #1).

The effects of the populations across the locations seem to rely on individual tests and PC analysis. It would seem to be possible to incorporate these tests more directly in the linear modeling analysis, and it isn't quite clear why this wasn't conducted.

We respond to this question below, in Recommendations for the Authors (first item from you).

I'm a bit puzzled by the discussion on how to find causative loci. This seems to focus solely on GWAS as the solution, with a goal to sequence vast individuals. But the loci that the manuscript discussed were found by a combination of structured mapping populations followed by molecular validation that then informed the GWAS. As such, I'm unsure if the proposed future approach of more sequencing is the best when a more balanced approach integrating diverse methods and population types will be more useful.

We are puzzled by this comment in return. Our statement about more sequencing (penultimate sentence of discussion) was referring to achieving a better understanding of the history of migration and selection rather than identifying causative loci.

**Recommendations for the authors:**

**Reviewing Editor Comments:**

The reviewers provide consistent and complementary recommendations that you should consider in a revision. In particular, you should try to better embed your results into the broader literature, both from Arabidopsis and other plant species. Also, try to make your text more accessible to readers outside the special topic.

Agreed!

**Reviewer #1 (Recommendations for the authors):**

(1) l.545: Here, the text states, importantly, that the accessions were randomized in the greenhouse. However, I have found no mention of this in the referenced Brachi et al. paper. I'm concerned that this be reported accurately and also that, if the accessions were not, in fact, randomized in the greenhouse, the potential for environmentally induced maternal influences to be confounded with genetically based differences be acknowledged, esp. in the case of the seed size difference found for B accessions.

Accessions were continuously randomized in the greenhouse although we stopped moving them when they flowered to reduce the risk of contamination. All the seeds for all the accessions were produced in the same greenhouse, in the same conditions, in one single planting. We state this clearly in the revised paper.

As for the seed size variation, note that the measurements we use in the paper were not taken on the seed we used in the experiments, so there is no reason they should be correlated with fitness if fitness was influenced by maternal effects. Moreover, we have plenty of data demonstrating that seed size variation is mostly genetic, and we added a supplementary figure showing that the measurements we used are strongly correlated with those of other experiments, including one done in the field. Finally, we already included a figure demonstrating that beach populations generally have much larger seeds.

(2) l.593: BLUPs are estimated with statistical uncertainty. I realize that it would be far from straightforward to take into account their sampling variances in the GWAS, but it should be acknowledged that ignoring the uncertainty of the BLUPs has an unknown impact on the findings from the GWAS.

All phenotypic measures come with error, and we had more replication than most GWAS studies (including essentially all human GWAS). The effect of such error is to reduce heritability and decrease the power of GWAS.

(3) l.48: As an earlier important reference demonstrating this point: Antonovics, Clay, Schmitt, 1987 Oecologia.

Indeed: added. Thanks!

(4) l.49: studied rapid evolution in a natural population[s] - delete 's'

Done.

(5) l.502: lead -> led

Corrected.

(6) l.53: The present study sought to gain insight into local adaptation in *A. thaliana*.; Vague, as is the rest of the intro.

Agreed. As noted above, the intro has been extensively reworked.

(7) l.533: What is alpha?

Alpha is the regularization parameter for the snmf algorithm. We added this clarification.

(8) l.553: Were the accessions also randomized in the field planting?

Yes, and we now state this.

(9) l.597: As I've noted in my public comments, the authors have done well to couch their genomic inferences with caveats. In line with such caution, I urge the authors to consider alternate phrasing to 'genetically determined' -> genetically influenced. Also l. 166: 'control of' -> influence on (among other instances).

No, “genetically determined” was correct on line 597, as this describes the model assumptions. But note that the liability-threshold model is by no means genetically deterministic: it is widely used in epidemiology to model genetic predisposition to diseases caused by the environment. For example, whether you get lung cancer or not primarily depends on luck and on your exposure to pollution (in particular smoking), but there are genes that influence how susceptible you are (to pollution; there are no genes that influence luck). There were some words missing in the sentence; hopefully things are clearer now.

Re l. 166, "control of” was changed to “effect on”, which is more accurate. Note, however, that there is nothing genetically deterministic here: the context is a variance-partitioning, and the results show very clearly that genetic factors play a minor role relative to environmental ones—and that most of the variance remains unexplained. (Luck?)

(10) l.595-602: Not clear.

As noted, some words were missing. Hopefully it is clearer now.

(11) l.744: Could have been confounded by cryptic native -- missing or extra word? Also next line.

Corrected.

(12) l.755-7: More direct phrasing would help readers understand this point.

Indeed. Sentence straightened out.

(11) l.129: 'The peak appears to involve a haplotype over 30 kb in length, which is consistent with a history of strong selection on this locus'. I don't understand the logic here. In what way should the length of the haplotype relate to the strength of selection? I would think this relates quite directly to the high degree of inbreeding.

Strong selection causes rapid allele-frequency change, leaving less time for recombination to break up associated haplotypes, causing increased linkage disequilibrium. This is standard population genetics (e.g., Maynard Smith and Haigh 1974). Inbreeding also causes increased haplotype sharing, but genome-wide. Telling them apart is difficult, hence we used “consistent”. However, Reviewer 3 points to the existence of a segregating inversion in this region, which is an even more likely explanation, and we focus on this in the revision.

(12) l.132: 'This suggests that the variation for slug damage seen in Figure 4 may be partly mediated by glucosinolate production.' I find the logic unclear here, as well.

“This” referred to several observations, which is poor English. We rearranged the sentences to make our meaning clearer.

(13) l.173: 'the accession-effect on fecundity' -> variation among accessions wrt fecundity.

We rewrote this clunky sentence.

(14) l.175: PCA unclear. Is this the same PCA described at l. 638? That is the only mention of PCA that I find in the methods, but I don't see that it connects here.

No this doesn’t refer to the same analysis. In line 175 we talk about a vanilla, textbook PCA: confronted with 200 fecundity estimates in 8 experiments, we used PCR to look for patterns across the 8 dimensions, and found that 3 dimensions captured most of the variation (as shown in the heatmap). We have added a few sentences about this in the methods. Line 638 describes the way images of plants were treated to estimate variation in rosette color.

(15) l.188: 'reveals what is causing them': Consider rephrasing to avoid language of causality, consistent with care taken elsewhere.

Well, the context here is very different: we are effectively doing a post hoc analysis, and there is nothing wrong with saying that “the significant value in the chi-square test is caused by an excess of…”, for example. The patterns we see in the PCA are caused by the kinds of patterns we go on to discuss—it captures these patterns, among other things. We changed “reveals” to “helps us understand” to be less biblical.

(16) l.219: downstream of -> conditional on.

Yes.

(17) l.235: Although native plants WERE growing nearby.

Yes.

(18) l.264: nearby: make this more explicit, i.e. within x km.

We changed the sentence to “Although native plants were growing within less than a hundred meters in most cases,...”

(19) l.285: none of our MAJOR conclusions depend on this.

We disagree. No conclusion in the paper could be confounded by potential natives.

(20) l.315: albeit it not as large as B accessions: delete 'it'.

Corrected.

(21) l.355: genetic basis of fitness -> genetic contribution to variation in fitness.

No, this is pretty much exactly Lewontin’s usage—the sentence (and section) is about changes in allele frequencies. The alternative suggested is less precise as we have no fitness estimates, and cannot say anything about variance.

(22) l.380-2: It seems to me that the authors could articulate a more compelling case.

Well, what we wrote is the truth. Of course all of this work also fits into a broader context, but those were the specific goals—and we achieved them. There are many papers that ask big questions, but actually answer much more limited ones.

(23) l.401: latitude vs. latitude???

Oops. Changed to “north vs. south”, which is hopefully less obscure!

(24) l.402: 'textbook local adaption,' -- the authors should acknowledge that rigid/simplistic thinking about LA has been recognized as a caricature for quite some time.

We do. Using “textbook” is meant to convey this: textbooks tend to be full of cartoonish simplifications, and most chapters on local adaptation will have a figure (cartoon or based on real data) showing reaction norms as two crossing lines.

(25) l.406: could maintain variation AMONG POPULATIONS [right?]

Could be either, but “among populations” is better in this context.

(26) l.419: Is there a possibility of a source envt maternal effect? See my comment on l.545.

As stated in response to the previous comment: In principle yes, but if so, then this environmental maternal effect happens to be strongly correlated with seed size, a trait we know to be genetically controlled and which is also extremely likely to influence seedling establishment. Experiments to confirm these results are underway—meanwhile we would be happy to accept bets against!

(27) l.434: 'very stable environments dating back to the last glaciation.' REF?

Ha! That would be Wikipedia references as the precise location of the “Littorina sea” and existence of obvious ancient beachlines now inland have entertained Swedish and Danish school children for generations. But the details are not important: the point is that beaches are vast (by *A. thaliana* standards) disturbed habitats maintained by the sea, and while they change, they do so on a geological time scale. Of course not all beaches harbor *A. thaliana*—the beaches of Hanö Bay are geologically unusual for Sweden—but this is beyond the scope of this publication. The sentence has been changed to something less specific making the relevant points.

(28) l.436: 'It is likely that the existence of S1 and S2 accessions is far more uncertain': unclear wording.

Yup. We rewrote the paragraph.

(29) l.446-7: 2nd person is jarring.

Reworded.

(30) ll.452-474: This is a welcome acknowledgement of the limits of molecular approaches to elucidating selection. It would be good scholarship to acknowledge earlier authors making such a case. I could suggest Rockman 2012. Evolution; Travisano and Shaw 2013. Evolution; Hoban et al. 2016. Am.Nat., and there are others.

True; added; thanks!

(31) l.490: 'might have to run a gauntlet of linkages to genes directly involved in local adaptation': This teleological wording is especially jarring.

(32) l.494: 'resistance allele to sweep': In concluding this manuscript, it does not seem appropriate to use as an example a single locus case. More broadly, I question the value/effectiveness of this paragraph as a conclusion to this manuscript.

We agree. The paragraph, gauntlets and all, has been replaced by discussion of why dissecting adaptive traits is hard.

**Reviewer #2 (Recommendations for the authors):**

(1) At the end of the introduction, the authors briefly outline the geographic scale of their study and mention both common garden and experimental evolution plots, but at this point in the manuscript, it is not clear how these differ, and as the methods only come at the very end, it would be better if some more details are provided here. Specifically, it could be mentioned explicitly here that common garden experiments consisted of placing greenhouse-grown plants in pots on the ground (but not burying/planting them), whereas selection experiments were established by sprinkling seeds in 1m 2 plots. This doesn't really become clear anywhere except in the methods, but this is fairly important for the interpretation of results.

We agree, and have expanded the description of the experiments in the legend to Figure 1, which also links to supplementary photos of the sites. We also note that the differences between the common-garden and the selection experiments should not be exaggerated. In particular, we did not use “greenhouse-grown plants”, but seedlings that had been allowed to establish outdoors in sheltered conditions, and we did not put “pots on the ground” but buried trays with holes in the bottom so that the plants could root in local soil. The main differences between the experiments are guaranteed establishment and lack of competition (from conspecific and other plants). This has also been clarified in the figure legend and in Methods.

(2) The authors discuss a difference in mortality between the two years of their common garden experiment and suggest that harsher winters could have been the cause of mortality in the north. However, they do not provide meteorological data to support this. Was the 2011-2012 winter harsher than the 2012-2013 winter, and did NM experience harsher conditions?

The problem is that we do not know what constitutes harshness from the point of view of the plant. Winters differ in many ways: temperature, duration, snow cover, etc. We have changed the relevant paragraph to make clear that our observations are consistent with those of Oakley et al., and that some aspect of winter weather is a plausible explanation.

(3) The authors find an indication for the involvement of the AOP cluster in overwinter survival, which, among other things controls the accumulation of hydroxyl/alkenyl/methylsulfinyl glucosinolates. Is the chemotype of the accessions in this study known?

Indeed they are! We had downloaded the data from Katz et al (2021), but the analysis did not make it into the first version of this paper. Thanks for the encouragement. We added a figure showing that their chemotypes are strongly associated with slug damage, supporting a causal relationship.

(4) L68: 'we added one field site in eastern Skane' - which site does this refer to? It seems odd to mention this before the common garden/selection experiments are mentioned. It should be clear that this is specifically referring to the latter.

Yes, this was out of place. The site is discussed later, when it becomes relevant.

(5) Figure 1: It would be useful if common garden and experimental evolution sites used different colors. In contrast, the use of colors for seasons in part C is unnecessary and distracting, as the red and blue colors for fall and winter are very similar to the colors for S1 and B.

Fixed.

(6) L90: The authors discuss mortality, but figures show survival. This seems an unnecessary complication for the reader, and the same unit should be used for discussion and presentation.

Survival is one minus mortality. We trust the readers to be able to do this conversion.

(7) L99: 'the converse was not true' - it is not immediately clear what this refers to. Referring to the non-linear relationship in panel 3a would make this clearer.

It refers to the result that the accessions with high mortality in NM did not necessarily have high mortality in NA. We think this is clear from the sentence in question.

(8) L108: 'S2 accession were also strongly affected' - this can be gleaned from the figures, but it is not immediately obvious as they are relatively complex. Could mean mortality/survival rates be provided to facilitate this?

We could but, throughout the paper, we have attempted to improve readability by not interrupting the text with numbers or repeating details that are presented in the figures.

(9) L117: 'however, an indirect association is likely' - what is meant by this?

We meant that it seems more likely that some accessions are more sensitive to stress regardless of source. This has been clarified.

(10) Figure 6: What are the two horizontal bars? What are dashed lines? Provide appropriate labels and a figure legend.

The top is a zoom-in of the bottom and the dashed lines outline the zoomed-in region. Clarified in legend.

(11) L174: Define 'BLUPS' here.

Done.

(12) L198: 'S1 and S2 accessions generally had higher fecundity in 2012-2013 than in 2011-2012' - this is not obvious from Figure 9. Especially for S1 (dark blue presumably), there appears to be no difference visible between years.

Changed text to note that differences are sometimes small, but that the stated pattern is seen in 13 out of 16 comparisons (which has p = 0.01 using a sign test).

(13) L401: 'latitude vs. latitude' - what is meant by this? Or is this a mistake?

Northern vs. southern. This has been clarified. Also noted by Reviewer 1.

(14) L436: 'the existence of S1 and S2 accessions is much more uncertain' - this is a bit of an odd expression. Could it be rephrased?

This has been clarified and the paragraph rewritten. Also noted by Reviewer 1.

(15) L673: Were plots tilled before sowing, or cleared of vegetation? If not, what other vegetation was present at sowing?

No clearing of vegetation was done except at the SR site, which was a weedy agricultural field. This and other information has been added. No vegetation surveys were made, but we added some more photos to give an idea of what the sites were like.

**Reviewer #3 (Recommendations for the authors):**

(1) In the section on lines 156-190 the large dataset is analyzed by accession in the linear model presented in Figure 6. Then, in Figure 8, the proposed populations appear to be tested individually in each experimental unit, leading to the probability argument in line 193. Is there a reason not to simply have accession nested within population in the model shown in Figure 6 as a way to directly test the between vs within population level components influencing the model? If the accessions are different, then it would be viable to treat this as random rather than fixed. Similarly, the field sites could be parsed into subsets as well.

Excellent question. We thought a lot about this. Fig. 6 presents a standard ANOVA that simply shows that the overall pattern is what we hoped for: very large effects of site, year, and accession, plus substantial interaction effects. This motivates the exploratory analysis presented in Figs. 7-9, where we focus on how the relative performance of accessions within experiments depended on the fixed effects (year and site) and whether this matched their (arbitrarily but independently defined) group designation—as would be expected under local adaptation.

We could explicitly add “group” to the model, as suggested, but this would give it a reality we do not think it deserves. Site, year, and accession are very much real, whereas group is the outcome of a somewhat arbitrary clustering of genotypes (i.e., accessions), analogous to race in human genetics, but without the sociological factors that sometimes warrant including race in a model not only because of direct genetic effects. Of course we could try to partition genotype and phenotype into within- and between-population variation using the classical quantitative genetics framework (i.e. Fst/Qst), but then we should have an a priori definition of “population”, which we don’t have. In addition to this fundamental objection, limited experimentation suggests that fitting a far more complex, nested mixed-effects model to our data is difficult in practice.

Thus we prefer our original approach. We have changed the writing to clarify our logic.

(2) Similarly, I'm not quite sure that the PC analysis is helping as the section is somewhat difficult to read given that the same impressions from Figure 7 are more explicitly shown in Figures 8 and 9.

Well, the PCA (Fig. 7) is what led us to the analyses in Figs 8-9, where we interpret the PCs in terms of group behavior. We have tried to clarify our logic in writing.

(3) Line 87-88 - An honest question, what is considered substantial divergence? The Fst values range from 0.05 to 0.27, suggesting that the divergences range across a spectrum and not all are substantial.

We removed the sentence. That the divergence is substantial enough to make GWAS difficult becomes clear later (although the extent to which this is due to selection rather than marker divergence is not clear).

(4) Line 129-130 - The 30kb AOP haplotype identified is likely the inversion associated with the major phenotypic variation identified in Sweden within Katz et al 2021. As such, the local LD structure may represent blocked recombination as much as linked selection.

Of course; we missed that. Thanks!

(5) Line 131-132 - I'm not quite sure what the evidence is that the MAM locus is more important.

The AOP and MAM loci are epistatic, and both have been found to have influence on fitness in Arabidopsis and Brassica ssp in the field, along with sequence signatures of selection for both loci in multiple Brassicaceae. I'm unsure if this statement is supported.

Indeed. This was a lazy and misleading reference to the fact that the MAM peak in Katz et al explains more of the variance than the AOP peak. It has been consigned to the dustbin of history. Instead we have added a figure showing that the glucosinolate profiles presented in that paper are highly correlated with slug damage in our study. Based on these results, I believe AOP explains more of the variation, but we leave pursuing this for those directly working on these pathways. The correspondence between our studies is certainly a nice confirmation of both.

(6) Line 131-132 - It should also be noted that the MAM locus is variable in Sweden, albeit having multiple independent haplotypes that convergently create the same phenotype. There is an indication from Gloss 2022 that these haplotypes may create small-effect phenotypic variation.

Correct again. What we meant to say was that the major polymorphism that is responsible for the highly significant MAM peak does not appear to segregate in Sweden, hence it is not surprising that we do not find an association at this locus either. We now say this. Needless to say, there could still be multiple variants at this locus that we do not have the power to detect.
